# Differential Expression of *Drosophila* Transgelins Throughout Development

**DOI:** 10.3389/fcell.2021.648568

**Published:** 2021-07-12

**Authors:** Katerina M. Vakaloglou, Maria Mouratidou, Athina Keramidioti, Christos G. Zervas

**Affiliations:** ^1^Center of Basic Research, Biomedical Research Foundation, Academy of Athens, Athens, Greece; ^2^Department of Biochemistry and Biotechnology, University of Thessaly, Larissa, Greece

**Keywords:** morphogenesis, cytoskeleton, actin-binding proteins, muscle, epithelia

## Abstract

Transgelins are a conserved family of actin-binding proteins involved in cytoskeletal remodeling, cell contractility, and cell shape. In both mammals and *Drosophila*, three genes encode transgelin proteins. Transgelins exhibit a broad and overlapping expression pattern, which has obscured the precise identification of their role in development. Here, we report the first systematic developmental analysis of all *Drosophila* transgelin proteins, namely, Mp20, CG5023, and Chd64 in the living organism. *Drosophila* transgelins display overall higher sequence identity with mammalian TAGLN-3 and TAGLN-2 than with TAGLN. Detailed examination in different developmental stages revealed that Mp20 and CG5023 are predominantly expressed in mesodermal tissues with the onset of myogenesis and accumulate in the cytoplasm of all somatic muscles and heart in the late embryo. Notably, at postembryonic developmental stages, Mp20 and CG5023 are detected in the gut’s circumferential muscles with distinct subcellular localization: Z-lines for Mp20 and sarcomere and nucleus for CG5023. Only CG5023 is strongly detected in the adult fly in the abdominal, leg, and synchronous thoracic muscles. Chd64 protein is primarily expressed in endodermal and ectodermal tissues and has a dual subcellular localization in the cytoplasm and the nucleus. During the larval–pupae transition, Chd64 is expressed in the brain, eye, legs, halteres, and wings. In contrast, in the adult fly, Chd64 is expressed in epithelia, including the alimentary tract and genitalia. Based on the non-overlapping tissue expression, we predict that Mp20 and CG5023 mostly cooperate to modulate muscle function, whereas Chd64 has distinct roles in epithelial, neuronal, and endodermal tissues.

## Introduction

Actin networks are fundamental cellular scaffolds that provide structural integrity in most cell types and modulate cellular contractility. A large fraction of intracellular actin is in the unpolymerized state, and the precise regulation of formation or dissociation of actin filaments is determined by the diverse function and subcellular localization of actin-binding proteins (Winder and Ayscough, 2005; Dominguez and Holmes, 2011; Pollard, 2016).

Transgelins encompass an actin-binding protein family, well-conserved from yeast to human, implicated in cytoskeleton remodeling (Shapland et al., 1988; Assinder et al., 2009; Liu et al., 2020). Transgelin’s name was coined by its ability to induce actin gelation *in vitro* (Shapland et al., 1993), while subsequent studies revealed actin filament bundling activity and its role in cellular contractility (Han et al., 2009). Transgelins are characterized by the presence of an N-terminal single calponin homology domain (CH) and a single C-terminal calponin-like repeat (CLR or CLICK repeat) (Assinder et al., 2009). In mammals, transgelin proteins are encoded by three genes that display differential tissue expression: (a) TAGLN (or SM22a) which is abundantly expressed in visceral and vascular smooth muscle cells (Lees-Miller et al., 1987; Lawson et al., 1997; Camoretti-Mercado et al., 1998; Assinder et al., 2009); (b) TAGLN2 (or SM22β) which is expressed in a wide variety of tissues and organs including smooth muscle cells, lung epithelium, gut, ovary, nephrons, pancreas, and T cells of the immune system (Zhang et al., 2002; Na et al., 2015; Meng et al., 2017; Yin et al., 2019). Interestingly, TAGLN2 elevated expression has been associated with progression of colorectal cancer (Zhang et al., 2010; Elsafadi et al., 2020); (c) TAGLN3 (also known as NP22 or NP25) is predominantly expressed in the nervous system (Ren et al., 1994; Depaz and Wilce, 2006).

There is a growing list of functional interplay between TAGLN and TGF-b signaling involved in human skeletal stem cells differentiation (Elsafadi et al., 2016), in polarized migration of mouse myofibroblasts at the leading edge of the ventral body wall (Aldeiri et al., 2017) and in reduced migration of human pulmonary arterial smooth muscle cells during hypoxia (Zhang et al., 2014). Additional studies have linked the elevated levels of TAGLN with the invasiveness of human hepatocellular tumorigenic cells (Lee et al., 2010) and with the inhibition of vascular smooth muscle cell proliferation *via* suppression of the Raf-1-MEK1/2-extracellular signal-regulated kinase 1/2 signaling pathway (Dong et al., 2010). Several studies indicate the colocalization of all three transgelin members with F-actin in certain cell types, namely, TAGLN in fibroblasts (Shapland et al., 1993), TAGLN2 in T-cell immunological synapse (Na et al., 2015), and TAGLN3 in neuroblastoma cells (Mori et al., 2004). Additional studies have also shown TAGLN presence in the nucleus (Bregant et al., 2009; Lin et al., 2009; Lew et al., 2020). Given the emerging importance of actin’s role inside the nucleus, the mystifying nuclear localization of transgelins could be related with actin-mediated effects on transcriptional regulation or nuclear integrity preservation (Ulferts et al., 2020). Due to the overlapping expression of TAGLN and TAGLN2 in mammals, genetic analysis has provided compelling evidence for the functional requirement of transgelins only in two examples: first, the requirement of TAGLN2 in T-cell immunological synapse for the stabilization of actin cytoskeleton (Na et al., 2015); second, the requirement of TAGLN in atherosclerosis by shifting the balance of smooth muscle cell contractility to proliferation during vascular remodeling (Zhang et al., 2001; Feil et al., 2004). Subsequent studies have shown that deletion of TAGLN was associated with a negative regulation of calcium-independent vascular contractility (Je and Sohn, 2007). Therefore, additional genetic studies are required to unravel specific transgelin requirement in several tissues in which they are expressed. *Drosophila* poses an excellent genetic model organism to evaluate the functional requirement of transgelins *in vivo*. In addition, the ability to perform high-resolution live imaging in the unfixed intact fly at various developmental stages allows the precise spatiotemporal characterization of fluorescently tagged proteins expressed from their endogenous regulatory elements (Kanca et al., 2017).

In *Drosophila*, there are also three genes encoding transgelin proteins: *Chd64* (*CG14996*), *mp20* (*CG4696*), and *CG5023*. The three fly transgelin proteins share an overall identity with their mammalian counterparts ranging from 44 to 53%. Chd64 displays higher identity for human TAGLN-3 and TAGLN-2 (42 and 41%, respectively), Mp20 for TAGLN-3 and TAGLN (42 and 41%, respectively), while CG5023 appears closer to Calponin 1 (42%), rather to the other three transgelins (36–39% for the transgelins). However, the overall CG5023 protein sequence falls in the transgelin protein family rather than to the calponins, which are not present in the fly genome. Mp20 was the first *Drosophila* transgelin which was identified to be expressed in a subset of adult muscles (Ayme-Southgate et al., 1989). Currently, the only available data regarding the expression of fly transgelins are derived from large-scale efforts to characterize *Drosophila* gene expression patterns (Tomancak et al., 2002; Weiszmann et al., 2009; Frise et al., 2010; Graveley et al., 2011; Brown et al., 2014). Of particular importance is the identification of both Mp20 and CG5023 in the list of genes whose expression in the females is modulated by male accessory gland proteins (Baker et al., 2007). To identify each transgelin protein’s functional requirement in *Drosophila* development, we have initially generated and characterized a variety of molecular and genetic tools and utilized them to thoroughly investigate the differential expression pattern of all three proteins during fly development. Such information will uncover potential functional redundancy between different transgelin members due to their overlapping expression and allow us to design a rationale genetic scheme to analyze specifically their function in distinct developmental stages and tissues in *Drosophila*.

Here, we report the expression pattern of all three transgelins in the developing fly and identify the subcellular localization of each protein *in vivo*. We have uncovered that Mp20 and CG5023 are expressed almost exclusively in the somatic and visceral musculature as well as the heart of the fly, while Chd64 is expressed specifically in epithelia and certain neuronal tissues. Based on our work, we predict that a double mutant of *Mp20* and *CG5023* is likely the optimum experimental genetic approach to identify transgelin-mediated functions in muscle cells and a single *Chd64* mutant to assess the involvement of transgelin in non-muscle tissues.

## Results

### Conservation of Actin-Binding Motifs in *Drosophila* Transgelins

Transgelins are considered actin-binding domain proteins containing two regions that confer actin-binding and/or actin bundling activity: the CLR region at the C-terminus (Fu et al., 2000) and the recently identified motif located between the CH-domain and the CLR (Na et al., 2015). We compared the protein sequence of all three *Drosophila* transgelins with their mouse and human homologs and confirmed the strong conservation of both CH-domain and CLR (Supplementary Figure 1A). We then analyzed the actin-binding motif (ABM) identified recently in mouse TAGLN2 (Na et al., 2015). The protein sequence alignment in this specific region indicated major differences between mammalian and fly transgelins (Supplementary Figure 1B). The mouse TAGLN2 motif **K**^153^KSKENP**R**^160^ retained only the first and the last positively charged residues in CG5023 (K^128^ and R^131^) and in Chd64 (K^148^ and R^151^). This is consistent with the lower identity in this motif displayed by the mouse TAGLN (50%) and TAGLN3 (75%). Finally, we utilized the online available tool cNLS Mapper^1^ (Kosugi et al., 2009) to identify a prediction value for bipartite nuclear localization signal. We identified a score above 4 for human and mouse TAGLN and TAGLN2 and all three fly transgelins, indicating that the protein can be localized both in the cytoplasm and nucleus (Supplementary Figure 1A).

### Expression Pattern of *Drosophila* Transgelins in Embryo

**FIGURE 1:**
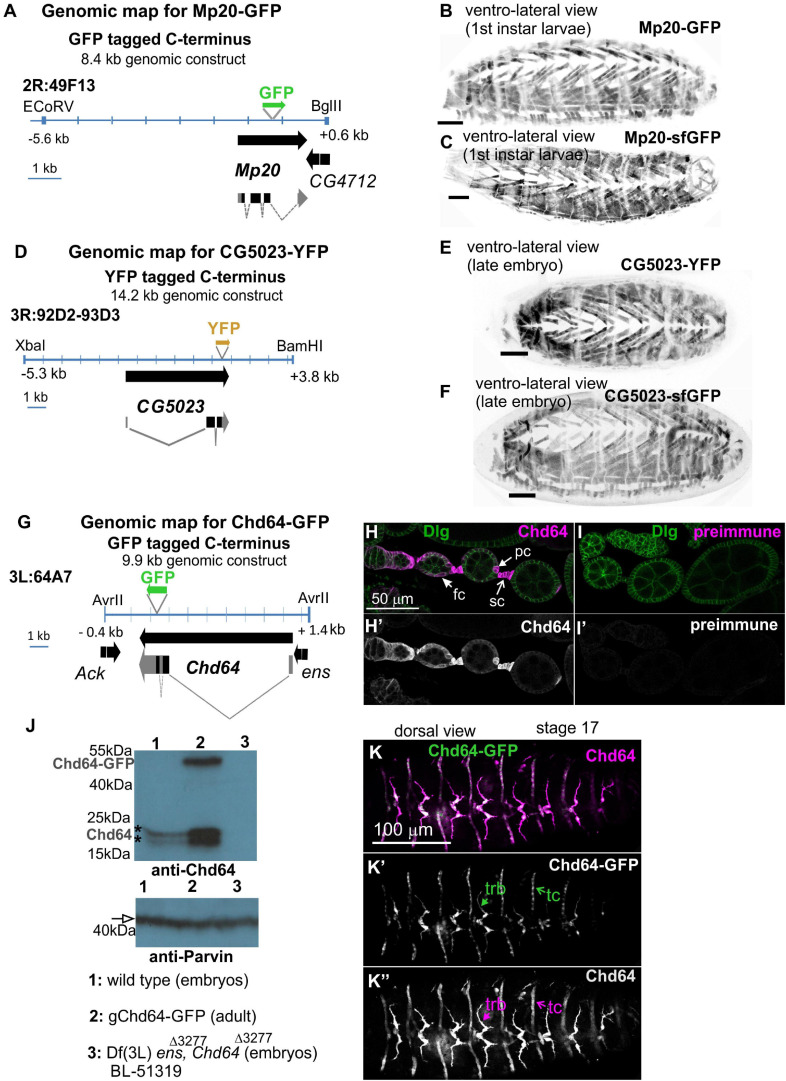
Embryo orientation: anterior-left, dorsal-up. Construction and characterization of genetic tools to study transgelin expression in *Drosophila*. **(A,D,G)** Engineered genomic fragments including GFP or YFP as translational fused reporters for **(A)** Mp20, **(D)** CG5023, and **(G)** Chd64. **(B,C,E,F)** Engineered genomic fragments drive expression comparably to the available fosmid library strains both for **(B,C)** Mp20 and **(E,F)** CG5023 proteins. **(H–K,H′,I′,J,K–K″)** Specificity of the Chd64 polyclonal antibodies is verified by **(H,I,H′,I′)** immunofluorescence of egg chambers and **(J)** Western blot analysis. **(H,I)** Egg chambers probed with antibodies against Chd64 and Disks Large (Dlg) to visualize the follicle epithelium. **(H,H′)** Chd64 was detected in polar cells (pc), follicle cells (fc), and stalk cell (sc), while **(I,I′)** egg chambers probed with a preimmune serum from the same animal did not show immunoreactivity. **(J)** Western blotting of protein lysates prepared from embryos (lanes 1 and 3) and adult expressing also the Chd64-GFP transgene (lane 2) probed with antibodies against Chd64 (top) and Parvin (bottom) to verify equal loading. The anti-Chd64 serum detected two close bands (indicated with the asterisks) just below the 25-kDa protein marker in lanes 1 and 2, one additional band corresponding presumably to the Chd64-GFP band at approximately 50 kDa in lane 2, but not in lane 3, because this protein lysate was prepared from embryos homozygous for the deficiency that removes the entire promoter region of the Chd64 locus. **(K–K″)** Late-stage embryo expressing Chd64-GFP (green) probed with an antibody against Chd64 (magenta). Both detected Chd64 and Chd64-GFP proteins coexpressed and colocalized in epidermal tendon cells (tc) and the tracheal branches (trb). Each image is representative of at least three different imaged embryos or egg chambers of the same genotype and markers used. Embryo orientation: anterior-left. Scale bar in panels **(B,C,E,F)** is 50 μm.

To investigate the expression pattern of all three transgelin proteins in *Drosophila*, we initially examined live embryos containing fluorescent translational reporters: GFP for *Chd64* and *Mp20* and YFP for *CG5023*. All engineered genomic fragments include the entire genomic region spanning each gene (see section “Materials and Methods” and Figures 1A,D,G). To further verify whether the engineered genomic regions include all necessary regulatory elements to drive transgelin expression, we additionally examined the available tagged flyfos TransgeneOme (fTRG) containing insertions for *Mp20* and *CG5023* tagged with sfGFP (Sarov et al., 2016). Both the engineered genomic translational reporters and the fosmid strains uncovered identical expression patterns (Figures 1B,C,E,F). These findings narrowed down the necessary genomic region that is sufficient to drive gene expression at the endogenous level for both genes: 8.8 kb for Mp20 and 14.2 kb for CG5023 (Figures 1A,D). Furthermore, we developed specific polyclonal antibodies for the full-length Chd64 protein. We verified the specificity of these polyclonal antibodies by three experimental approaches. First, we performed immunofluorescence stainings in egg chambers (Figure 1H). Preimmune serum from the same animal resulted in complete absence of immunoreactivity (Figure 1I). Second, we performed Western blot analysis in both embryonic and adult lysates from a strain expressing the engineered transgene Chd64-GFP. As a control, we utilized a genetic strain lacking the genomic region spanning the entire promoter region of Chd64 and resulted in total absence of the detected Chd64 endogenous protein band (Figure 1J). Third, Chd64-GFP expression from the 9.9-kb genomic fragment was compared with endogenous Chd64 detected with Chd64 antiserum in late-stage embryo (Figures 1K–K″). Endogenous Chd64 protein was largely colocalizing with Chd64-GFP in identical tissues (Figures 1K–K″). Thus, we are confident that we have truly uncovered the endogenous developmental expression profile for each *Drosophila* transgelin protein as we demonstrate below.

#### Mp20 and CG5023 Expression

**FIGURE 2:**
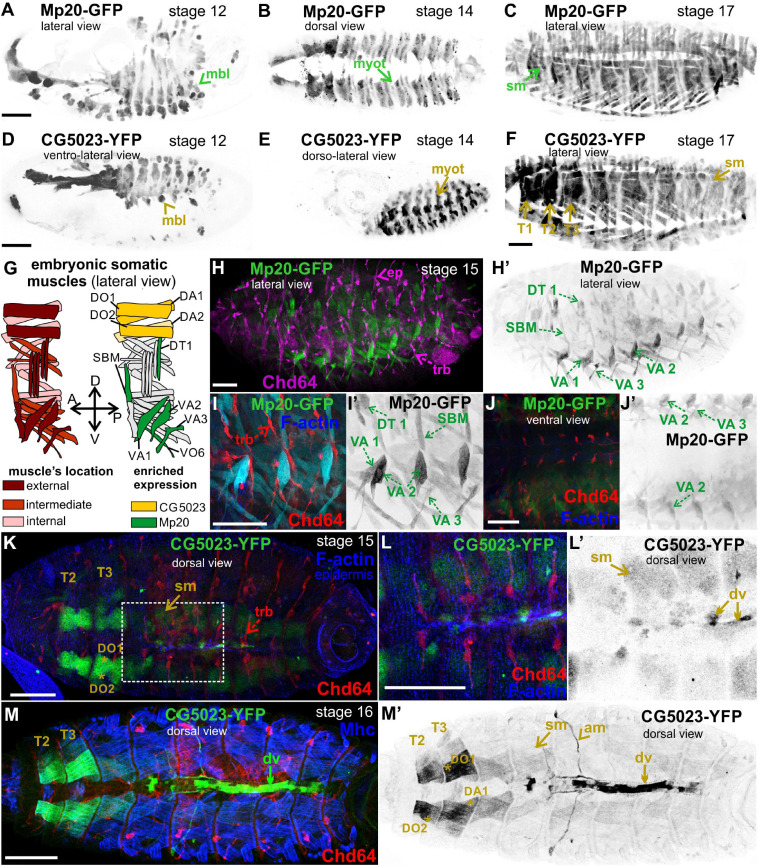
Mp20 and CG5023 accumulation in the developing embryo. Confocal micrographs of embryos expressing fluorescently tagged transgelin proteins that reveal their endogenous tissue distribution at various developmental stages. **(A–F)** Living embryos expressing **(A–C)** Mp20-GFP and **(D–F)** CG5023-YFP. **(A,D)** Mp20 and CG5023 are expressed in all myoblasts at stage 12, **(B,E)** in all segmented organized myotubes at stage 14, and **(C,F)** in the entire somatic musculature at stage 17. **(G)** Schematic representation of the somatic muscle pattern (lateral view) in an abdominal segment of a late-stage embryo. **(H–J)** Stage 15 fixed embryos expressing Mp20-GFP protein, probed against Chd64 to verify their distinct patterns of expression in mesodermally and ectodermaly derived cells, respectively. Mp20-GFP specifically accumulates in DT1, VA1, VA2, VA3, and SBM abdominal muscles. **(I,J)** High magnification of embryonic muscles to visualize the muscle distribution and enrichment of Mp20 in muscles DT1, VA1–3, SBM, and DT1 and tracheal branches intense accumulation of Chd64 (red); F-actin was labeled with Alexa 647-phalloidin (blue). **(K–M)** Late-stage fixed embryos expressing CG5023-YFP, probed with antibodies against Chd64 (red) and muscle myosin (blue). **(K,L)** In stage 15 embryo, CG5023-YFP was significantly elevated in DO1/DA1 somatic muscles within T1/T2 thoracic segments and clearly apparent in the dorsal vessel. **(M,M′)** In stage 16 embryo, high levels of CG5023-YFP were detected in DO1/DA1 muscles within thoracic segments and also in the alary heart muscles along with elevated accumulation in the dorsal vessel. Each image is representative of at least five different imaged embryos of the same genotype and markers used. mbl, myoblast; myot, myotube; sm, somatic muscles; T1–T3, thoracic segments 1, 2, 3; ep, epidermis; trb, tracheal branch; DT, dorsal transverse; SBM, segment border; DA, dorsal acute; VA, ventral acute; VO, ventral oblique; dv, dorsal vessel; am, alary muscles. Scale bars: 50 μm in all panels.

Both Mp20-GFP and CG5023-YFP were initially detected in myoblasts during germ band retraction (Figures 2A,D). From our live-imaging observations, we could clearly identify the expression of CG5023-sfGFP in all myoblasts—with variable levels of expression—prior to myoblast fusion (Supplementary Figure 2). As embryogenesis proceeds, both Mp20-GFP and CG5023-YFP were detected in the migrating myotubes (Figures 2B,E). At stage 15 of embryogenesis, Mp20-GFP was significantly elevated in abdominal muscles VA2 (fluorescent mean intensity, FMI = 194.8), VA1 (FMI = 106), VA3 (FMI = 102.3), DT1 (FMI = 96.8), and SBM (FMI = 102.8) vs. the other somatic muscles (FMI = 35) (FMI was measured in seven embryos) (Figures 2G,H–J). CG5023-YFP was accumulated at significantly higher levels in the thoracic muscles (DO1/DA1 and in muscles DO2/DA2 within the T2–3 segments (FMI = 163.5) vs. the other somatic muscles (FMI = 42.6) (FMI was measured in eight embryos) (Figures 2K–M). Additionally, at the end of embryogenesis, both Mp20-GFP and CG5023-YFP were accumulated in all somatic muscles (Figures 2C,F) and in the dorsal vessel (Figures 2M,M′). CG5023-YFP was additionally expressed in the alary muscles of the heart (Figures 2M,M′).

#### Chd64 Expression

**FIGURE 3:**
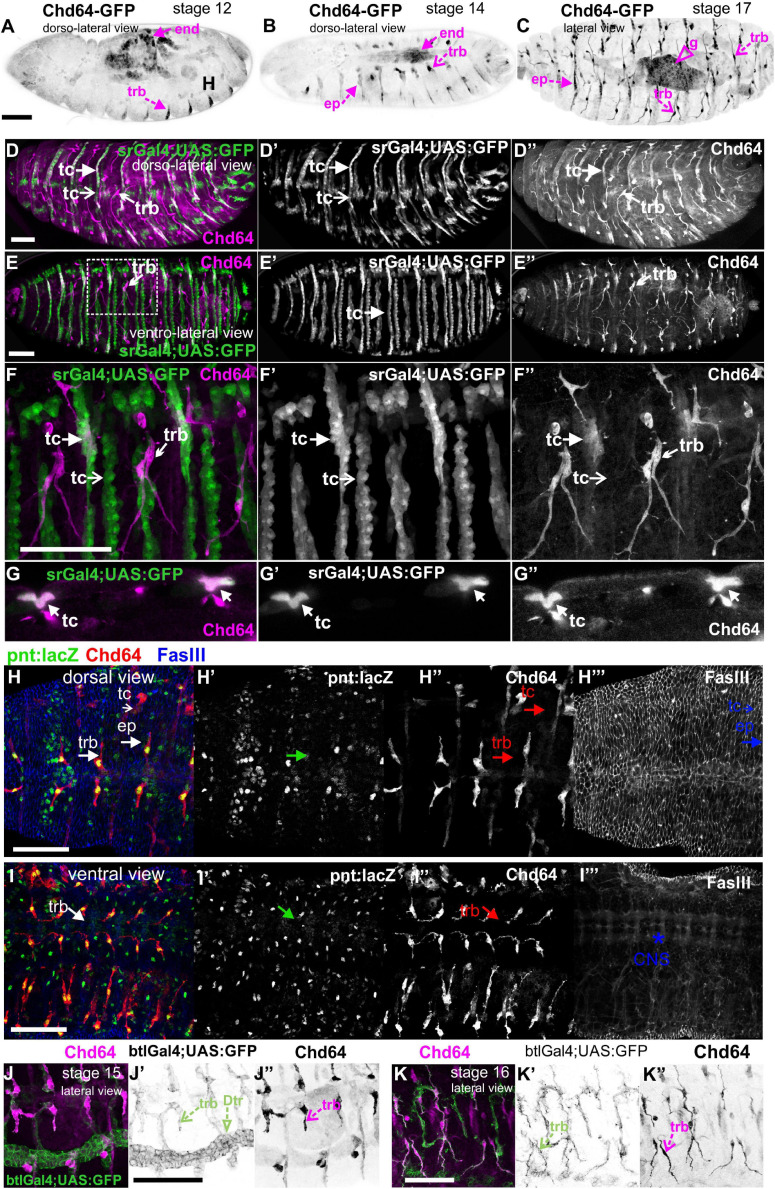
Chd64 protein accumulation in the developing embryo. **(A–C)** Living embryos expressing Chd64-GFP. **(A)** In stage 12 embryo, Chd64-GFP is detected in gut primordial cells and epidermally associated structures localized at the segment borders. **(B)** In stage 14 embryo, Chd64-GFP further accumulates in the alimentary track and epidermally associated structures localized at the segment borders. **(C)** In late stage 17 embryo, Chd64-GFP protein is heavily enriched in the gut, tracheal branches, and epidermal cells in a similar identified pattern. **(D–F)** Different embryos viewed dorsolaterally **(D)** and ventrolaterally **(E,F)**, expressing UAS:GFP under the *stripeGal4* which marks the tendon cells and probed with an antibody against Chd64. **(D)** A higher number of tendon cells located in the dorsal side of the embryo express Chd64 and colocalize with GFP. **(E)** In the ventral side of the embryo, the number of tendon cells expressing Chd64 is significantly lower. **(F)** The reduced number of tendon cells expressing Chd64 is clearly illustrated in the high-magnification image. Tracheal branches are evident in all views of the embryo and clearly depicted in the high-magnification image in panel **(F)**. **(G)** Horizontal single optical section of an embryo, to clearly demonstrate the expression of Chd64 in tendon cells. **(H–H′″)** Dorsal and **(I–I′″)** ventral view of stage 15 fixed embryo expressing the trachea-specific marker *pointedlacZ* (green) and probed against Chd64 (red) and Fasciclin III (blue) proteins. **(H′,I′)** *pointedlacZ* expression marks the nuclei of tracheal branches and colocalizing with Chd64 detected protein **(H″,I″)**; FasIII labels the **(H′″)** epidermis and **(I′″)** CNS. **(J,K)** Fixed embryos probed against Chd64 along with ectopic expression of btl-Gal4; UAS-GFP to highlight tracheal branches. **(J–J″)** In stage 15 embryo, the newly formed secondary branches of trachea marked by GFP expression are characterized by significant accumulation of Chd64 protein. **(K–K″)** In stage 16 embryo, Chd64-GFP is clearly accumulated in the long extensions of tracheal branches. Each image is representative of at least three different imaged embryos of the same genotype and markers used. end, endoderm; ep, epidermis; g, gut; trb, tracheal branch; tc, tendon cell; Dtr, dorsal trunk; CNS, central nervous system. Embryo orientation: anterior-left, dorsal-up. Scale bars: 50 μm.

**FIGURE 4:**
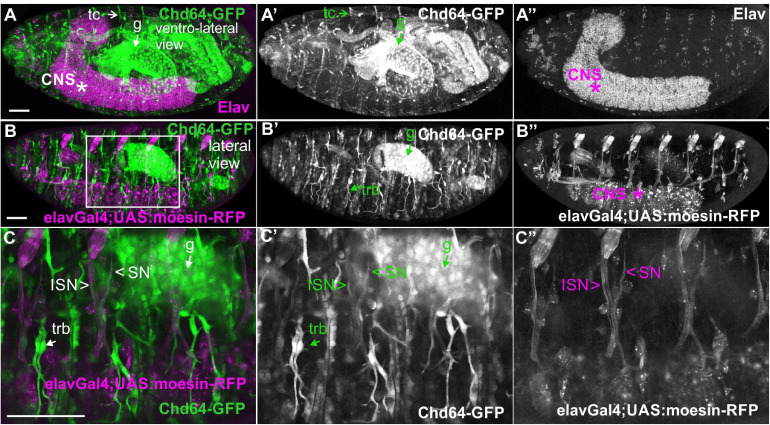
Chd64-GFP protein accumulation in certain neuronal cells in the embryo. **(A–C,A′–C′,A″–C″)** Confocal micrographs of fixed late-stage 16 embryos expressing Chd64-GFP and **(A–A″)** probed with antibodies against Elav or **(B–B″,C–C″)** expressing UAS:moesin-RFP in the CNS (under the *elavGal4*). **(C–C″)** High magnification of the boxed region in panel **(B)** demonstrates the low Chd64 expression levels in motor neurons and peripheral neurons during embryogenesis. Each image is representative of at least four different imaged embryos of the same genotype and markers used. trb, tracheal branch; tc, tendon cell; CNS, central nervous system; g, gut; ISN, intersegmental nerve; SN, segmental nerve. Scale bars: 50 μm.

We examined living embryos expressing the genomic translational fusion Chd64-GFP construct. The zygotically expressed Chd64-GFP protein appears concentrated in the presumptive region of the gut (Figure 3A) and strongly accumulates in the endodermal cells of the entire gut as embryogenesis proceeds (Figures 3B,C). We then directly compared Mp20-GFP/Chd64 (Figures 2H–J) and CG5023-YFP/Chd64 (Figures 2K–M) expression patterns. We detected Chd64 in epidermal cells only in the vicinity of the segment borders including tendon cells (Figures 3C–G). The latter were verified by the ectopic expression of UAS:GFP under the *stripeGal4*, which marks specifically tendon cells (Lee et al., 1995; Subramanian et al., 2003; Figures 3D–G). Furthermore, we utilized the trachea-specific markers *btlGal4* (Ghabrial et al., 2011) and *pointedLacZ* and verified that Chd64 was present mainly in the secondary and final tracheal branches but hardly detectable in the dorsal trunk (Figures 3H–K). Lastly, we found that during embryogenesis, Chd64 was expressed at relatively low levels in motor neurons and peripheral neurons (FMI = 60.5) (FMI was measured in six embryos) (Figures 4A–C).

### Expression Pattern of *Drosophila* Transgelins in Larvae and Imaginal Disks

**FIGURE 5:**
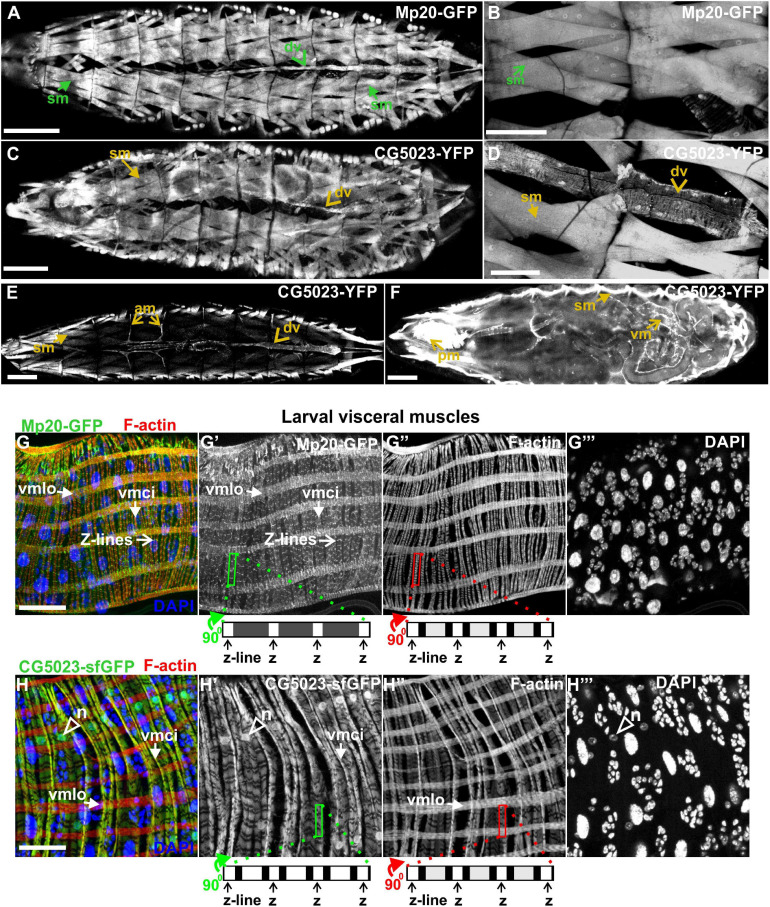
Mp20 and CG5023 protein expression in somatic and visceral muscles in larvae. **(A,B)** Mp20-GFP is strongly expressed in the full range of somatic musculature along with elevated levels in the dorsal vessel. Likewise **(C–E)** CG5023-YFP expression pattern demonstrates an endogenous distribution identical to Mp20-GFP in all somatic muscles and dorsal vessel. **(F–H)** CG5023-YFP, **(F)** Mp20-sfGFP, **(G–G″)** and CG5023-sfGFP **(H–H″)** proteins are preferentially expressed in the circumferential muscles of the gut. **(G–G″)** Mp20-sfGFP in both circular (vmci) and longitudinal (vmlo) visceral muscles. **(F,H–H″)** CG5023-YFP and CG5023-sfGFP solely in circular visceral muscles labeling them in a striated pattern along with a rather intense accumulation in their nuclei. Each image is representative of at least three different imaged larvae of the same genotype and markers used. sm, somatic muscle; dv, dorsal vessel; am, alary muscles; vm, visceral muscles; pm, pharyngeal muscles; vmlo, longitudinal visceral muscles; vmci, circular visceral muscles; n, nucleus. Scale bars: 100 μm in panels **(A–F)** and 25 μm in panels **(G,H)**.

**FIGURE 6:**
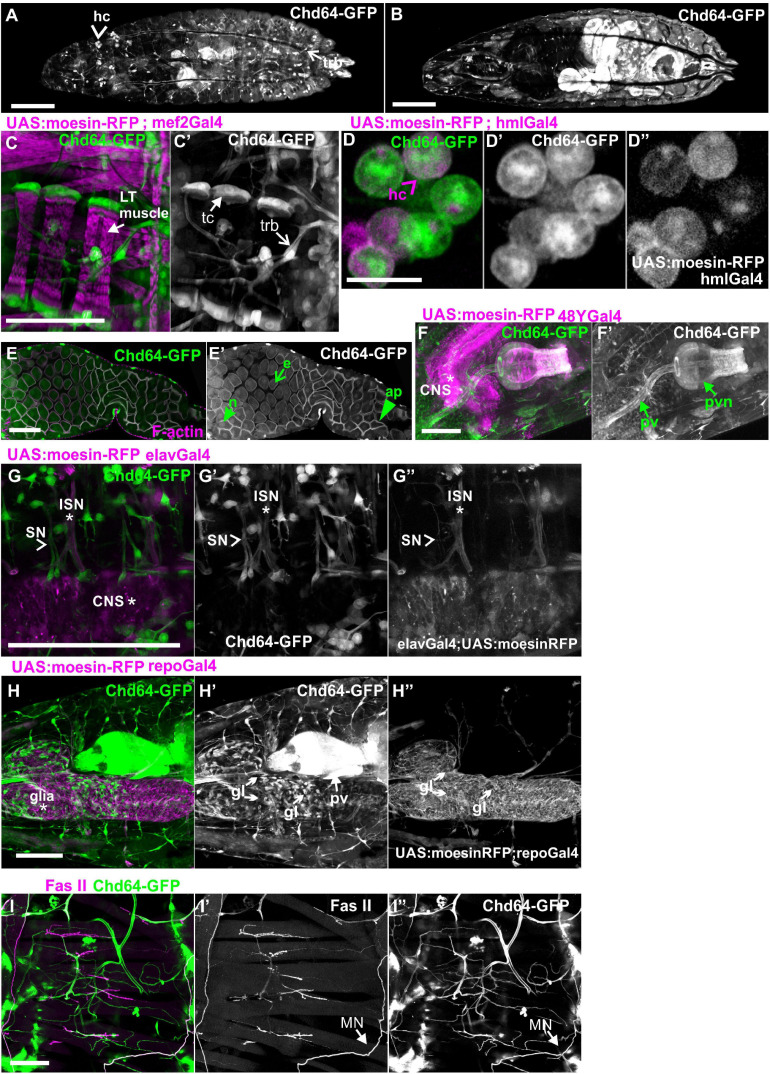
Chd64-GFP protein expression in several cell types in larvae. **(A,B)** First-instar larvae expressing Chd64-GFP. **(A)** Confocal projection of optical sections derived from the external dorsolateral side of the larvae to show endogenous expression in hemocytes and tracheal branches. **(B)** Confocal projection of optical sections derived from the interior part of the same larvae to show the intense expression of Chd64-GFP in the gut. **(C–I)** Chd64-GFP endogenous expression in several cell types including **(C,C′)** tracheal branches and tendon cells at the tips of the LT muscles labeled with the expression of *UAS:moesin-RFP* under the *mef2Gal4*; **(D–D″)** hemocytes labeled with *UAS:Moesin-RFP* driven by *hmlGal4*; **(E,E′)** enterocytes in the midgut area labeled also with F-actin, which is enriched apically together with Chd64-GFP; **(F,F′)** endodermally originating cells along the gastrointestinal tract in the proventriculus area, labeled with *UAS:Moesin-RFP* driven by *48YGal4*; **(G–G″)** motor neurons labeled with *UAS:Moesin-RFP* driven by *elavGal4*; **(H–H″)** glia cells residing in the CNS labeled with *UAS:Moesin-RFP* driven by *repoGal4*; and **(I–I″)** peripheral neurons innervating somatic muscles labeled with FasII. Each image is representative of at least four different imaged larvae of the same genotype and markers used. hc, hemocytes; LT, longitudinal; trb, tracheal branch; tc, tendon cells; pv, proventriculus; pvn, proventricular nerve; CNS, central nervous system; ISN, intersegmental nerve; SN, segmental nerve, gl, glia. Scale bars: 100 μm in all panels, except **(D)**: 5 μm.

Both Mp20-GFP and CG5023-YFP or CG5023-sfGFP maintain their strong expression in all body wall muscles (FMI = 200.5), dorsal vessel (FMI = 120.3), and alary muscles in the larvae (FMI was measured in seven larvae first–third instar) (Figures 5A–E). Moreover, we detected both Mp20-GFP and CG5023-YFP in the gut’s circumferential muscles (Figures 5F,G,H). Mp20-GFP is expressed in both longitudinal visceral muscles (vmlo) and circular visceral muscles (vmci) (Figures 5G–G″), while CG5023-sfGFP is expressed only in the vmci (Figures 5H–H″). Interestingly, CG5023-sfGFP localized both in a striated cytoplasmic pattern and also in the nucleus (Figure 5H). Chd64-GFP expression in larvae is maintained in secondary tracheal branches (Figures 6A,B), tendon cells (Figures 6C,C′), and circulating hemocytes (Figure 6A). The latter cell population was marked by *hmlGal4* (Charroux and Royet, 2009)-driven expression of *UAS:moesin-RFP* (Figures 6D–D″). Chd64-GFP was heavily accumulated in all endodermal cells of the gut (Figures 6E,E′) localizing both in the cytoplasm and the nucleus and further colocalizing with F-actin in the apical side of the epithelium. We found enriched concentration of Chd64-GFP in the proventriculus (Figures 6F,F′), and the entire gut epithelium was marked by *48YGal4*-driven expression of *UAS:moesin-RFP* (Figures 6F,F′) and the proventricular nerve (Figures 6F,F′). Finally, we identified elevated levels of Chd64-GFP in several neuronal cell types, including peripheral neurons (Figures 6G–G″), glia cells in the ventral nerve cord (vnc) (Figures 6H–H″), and motor neurons innervating somatic muscles (Figures 6I–I″).

**FIGURE 7:**
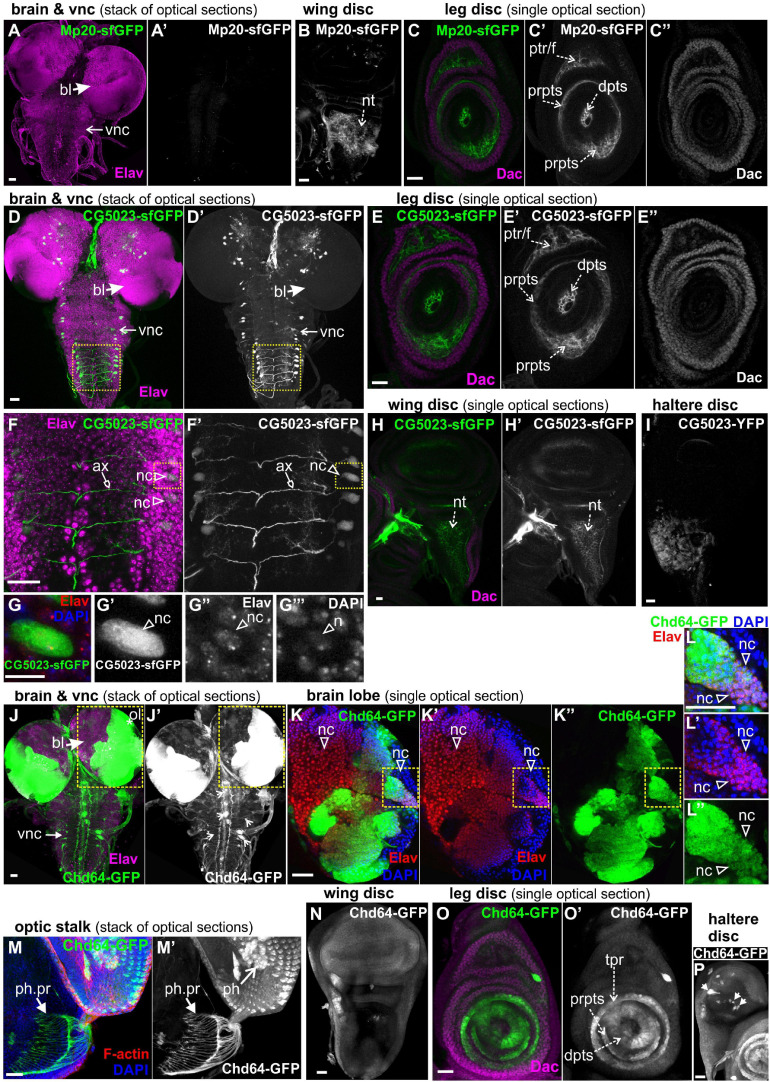
GFP-tagged transgelin protein expression in imaginal disks derived from third-instar larvae. Confocal micrographs of dissected disks expressing fluorescently tagged transgelin proteins and probed with various antibodies to label specific disk markers. **(A–C)** Mp20-sfGFP expression pattern. **(A)** Mp20-sfGFP is not expressed in the larval brain or ventral nerve cord, which both are labeled with Elav. **(B,C)** Single optical sections of the imaged disks. **(B)** Mp20-sfGFP is expressed in the wing disk notum region. **(C)** Mp20-sfGFP is expressed in specific regions of leg disks. Mp20-sfGFP is partially colocalized with Dachshund (Dac) in the progenitor’s area of trochanter and femur, but is additionally expressed in the distal tarsal segments, where Dac is not expressed. **(D–G)** CG5023-sfGFP protein expression pattern. **(D,D′,F,F′,G–G″′)** CG5023-sfGFP is expressed in certain neuronal cells both in the brain lobes and the ventral nerve cord (vnc), which both are labeled with Elav. **(F,F′)** High-magnification image of the boxed region in panels **(D,D′)**, where it clearly shows the coexpression of CG5023-sfGFP and Elav in the neuronal bodies residing laterally of the ventral midline. CG5023-sfGFP also accumulates in the axons. **(G–G″′)** The boxed region in panels **(F,F′)** was cropped and magnified to clearly demonstrate the colocalization of CG5023-sfGFP with the Elav-labeled neuronal cell within the nucleus marked by DAPI. **(E–E″)** Single optical section of leg disk expressing CG5023-sfGFP in a pattern reminiscent of what was above described for Mp20-sfGFP. CG5023-sfGFP is partially colocalized with Dac in the progenitor’s area of trochanter and femur, but is additionally expressed in the distal tarsal segments, where Dac is not expressed. **(H,H′)** CG5023-sfGFP is expressed in the notum region of the wing disk and additionally **(I)** in the haltere notum area. **(J–P)** Chd-GFP expression pattern. **(J–L)** Chd64-GFP is strongly expressed in the optic brain lobe pair, which was labeled with Elav. **(J,J′)** The prominent expression of Chd64-GFP was identified in the vnc area, but it was not colocalized with Elav which ruled out the neuronal identity of these particular cells. **(K–K″)** High magnification image of the boxed region in panels **(J,J′)** depicting one almost entire brain lobe. The image is one optical section derived from the middle part of the brain lobe to visualize the intense expression of Chd64-GFP in certain areas of the brain. **(L–L″)** The boxed region of **(K–K″)** was cropped and magnified to clearly demonstrate the coexpression of Chd64-GFP with the Elav in certain neuronal cells within the brain lobes. **(M,M′)** Chd64-GFP expressing eye-antenna disk probed for F-actin. Nuclei are marked by DAPI staining. Chd64 protein preferentially labels both the photoreceptor cells as well as the photoreceptor nerve terminals that end up in the optic lobe within the larval brain. **(N)** Chd64-GFP is expressed throughout the epithelium of the wing imaginal disk. **(O,O′)** Chd64-GFP is strongly accumulated in certain leg disk regions, where it is colocalized with Dac only in the progenitor’s area of tibia and first tarsal segments. Chd64-GFP is additionally expressed in the distal parts of the other tarsal segments. **(P)** Chd64-GFP is expressed in scattered cluster of cells all over the haltere pouch. Each image is representative of at least 10 different imaged larval imaginal disks of the same genotype and markers used. nc, neural cell; bl, brain lobe; vnc, ventral nerve cord; nt, notum; prpts, proximal primordium of tarsal segments; dpts, distal primordium of tarsal segments; tpr, tibia primordium; ptr/f, primordium of trochanter/femur; ol, optic lobe; ph, photoreceptor; ph. pr, photoreceptor projection. Scale bars: 25 μm, except panels **(G–G′″)**: 10 μm.

To study further the expression of all three transgelins in the transition from larvae to pupae, we examined imaginal disks derived from late third-instar larvae expressing fluorescent translational reporters for each *Drosophila* transgelin (Figure 7). Both Mp20-sfGFP and CG5023-sfGFP were expressed in relatively low levels in the imaginal disks. Mp20-sfGFP was not detected in the larval brain (Figure 7A), but it was detected in the notum region of the wing disk (Figure 7B). Based on Dachshund expression pattern (Mardon et al., 1994), we detected Mp20-sfGFP in the leg disk primordium of trochanter, femur, and tarsal segments (Figures 7C–C″). CG5023-sfGFP was detected in subsets of neuronal cells in the brain lobes and the vnc as illustrated with the Elav marker (Figures 7D,F,G); in the leg disk primordium of trochanter, femur, and tarsal segments (Figures 7E–E″); in the notum of the wing disk (Figures 7H,H′); and in the distal region of the haltere disk (Figure 7I). On the contrary, Chd64-GFP was heavily expressed in optic lobes and several other neuronal cells at the brain lobes (Figures 7J–L). Chd64-GFP was also found at the nerve terminals of the photoreceptors that end up in the visual lobe of the larval brain (Figures 7M,M′). Furthermore, Chd64-GFP was expressed in the entire epithelium of the wing imaginal disks (Figure 7N), in the leg disk primordium of tibia and tarsal segments (Figures 7O,O′), and a group of scattered cells in the haltere disk (Figure 7P).

### Expression Pattern of *Drosophila* Transgelins in the Adult Fly

**FIGURE 8:**
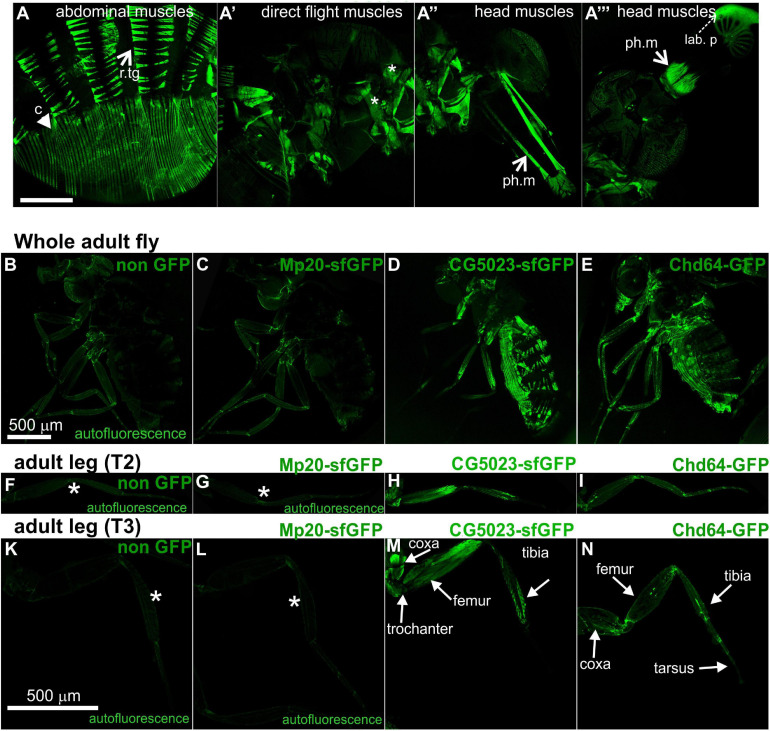
Transgelin protein expression in *Drosophila* adults. **(A–N)** Confocal micrographs of isolated adult tissues expressing fluorescently tagged transgelin proteins that reveal their endogenous pattern of distribution. **(A–A′″)** Adult fly expressing CG5023-YFP in panel **(A)** all the abdominal muscles, **(A′)** in a subset of thoracic muscles (asterisks), and **(A″,A′″)** both the pharyngeal and labial pulp muscles in the head. **(B–E)** Confocal micrographs of living adult flies imaged under identical settings to demonstrate, **(B)** tissue autofluorescence, **(C)** absence of Mp20-GFP in adult tissues, **(D)** expression of CG5023-sfGFP in abdominal muscles, and **(E)** Chd-GFP expression in various internal organs. **(F–I,K–N)** Confocal micrographs of isolated adult **(F–I)** T2 and **(K–N)** T3 legs. CG5023-sfGFP displays robust fluorescence signal in specific indicated parts of the adult legs. The strain used in panels **(B,F,K)** was *w,f* and did not express a GFP-tagged gene. Each image is representative of at least six different imaged adult flies and isolated adult legs of the same genotype. r.tg, retractors of tergites; c, compressors of abdomen; ph.m, pharyngeal muscles; lab p, labial pulp. Scale bars: 500 μm.

**FIGURE 9:**
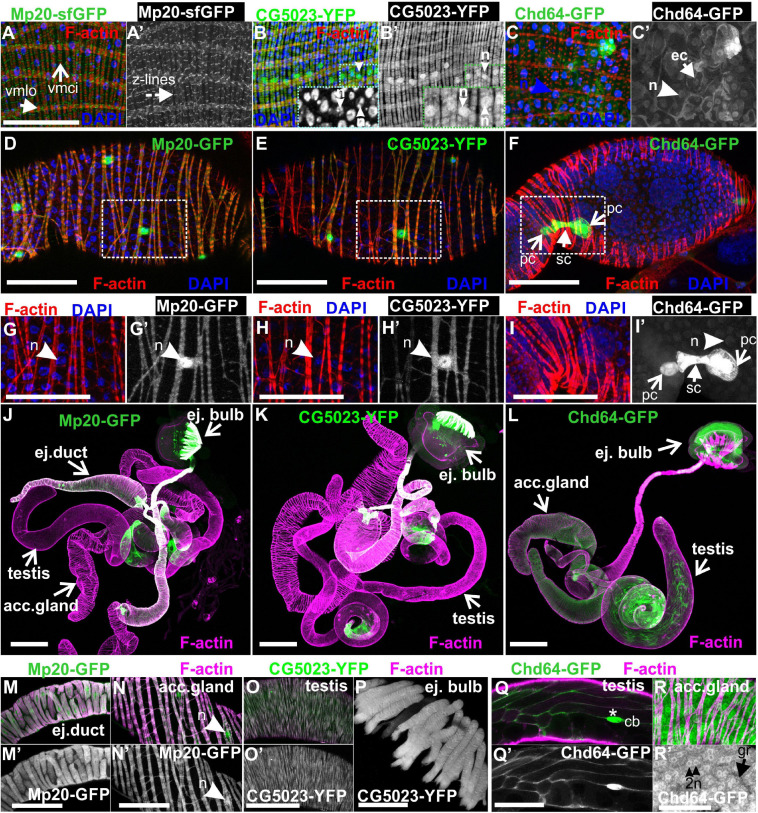
Transgelins expression in adult gut and genitalia. **(A,A′,B,B′,C,C′)** Tangential confocal sections of adult midgut regions expressing Mp20-sfGFP, CG5023-YFP, and Chd64-GFP. **(A,A′)** Mp20-sfGFP expression is observed in the Z-lines of both visceral circular and longitudinal muscle layers (vmci and vmlo respectively), whereas **(B,B′)** CG5023-YFP accumulates solely in the vmci showing nuclear localization along with colocalization to filamentous actin. **(C,C′)** Chd-GFP intestinal subcellular localization appears prominent in both the nucleus and cytoplasm of enterocytes. **(D–F)** Mp20-GFP, CG5023-YFP, and Chd64-GFP expression pattern in egg chambers of adult female genitalia. **(D)** Mp20-GFP and **(E)** CG5023-YFP are both expressed in muscle sheath surrounding the egg chambers, whereas **(F)** Chd64-GFP localizes in polar and follicle cells. **(G–I,G′–I′)** High magnification egg chambers expressing **(G,G′)** Mp20-GFP and **(H,H′)** CG5023-YFP both showing considerable nuclear localization and circular visceral muscle accumulation, while **(I,I′)** Chd64-GFP accumulation is specific to polar and follicle cells. **(J–L)** Mp20-sfGFP, CG5023-YFP, and Chd64-GFP expression pattern in multiple regions of adult male genitalia. **(M–R)** High magnification of male genitalia compartments expressing **(M,N,M′,N′)** Mp20-sfGFP and **(O,O′,P)** CG5023-YFP both apparent in **(M,M′)** the ejaculatory duct muscle sheath, **(N,N′)** the accessory gland muscle sheath, **(O,O′)** the smooth muscles surrounding testes and **(P)** the ejaculatory bulb muscle fibers. **(Q,R and Q′-R′)** Chd64-GFP expression in distinct cell types of male genitalia, particularly in **(Q,Q′)** the testis, as well as in **(R,R′)** the binucleated accessory gland epithelium. **(R,R′)** Chd64-GFP subcellular localization appears both nuclear **(R′)** and cytoplasmic **(R′)** in either type of these cells. Each image is representative of at least 4 different imaged adults or adult tissues of the same genotype and markers used. vmlo, longitudinal visceral muscles; vmci, circular visceral muscles; pc, polar cell; fc, follicle cell; ej. duct, ejaculatory duct; acc. gland, accessory gland; ej. bulb, ejaculatory bulb; acc. gland, accessory gland; cb, cystic buldge; gr, granule; n, nucleus Scale bars: 100 μm.

Both CG5023-YFP and CG5023-sfGFP were expressed in all abdominal muscles of the adult fly (Figures 8A,D). In the thorax, CG5023-YFP and CG5023-sfGFP were expressed only in a subset of muscles. Based on topology, we concluded that they were likely direct flight muscles (Figure 8A′). Moreover, CG5023-sfGFP was identified in the coxa, trochanter, femur, and tibia leg muscles (Figures 8D,H,M); in the pharyngeal muscles (Figure 8A″); and in the labial pulp (Figure 8A′″). In contrast, Mp20-sfGFP was not detected in the adult body muscles, including the thorax, abdomen, and leg. To verify this conclusion, we examined the fluorescence intensity of adult flies of a strain without endogenous expression of GFP and found low levels of autofluorescence comparable to the fluorescence intensity obtained in the Mp20-sfGFP flies (Figures 8B,C,F,G,K,L). The detection of Chd64-GFP in the whole adult fly was not really informative by confocal microscopy, but we certainly detected high levels of fluorescence intensity presumably derived from internal organs (Figures 8E,I,N). Consistent with a previous study (Ayme-Southgate et al., 1989), Mp20-GFP was also identified both in longitudinal (vmlo) and circumferential (vmci) visceral muscles of the entire adult gut (Figures 9A,A′). CG5023-YFP was expressed only in the vmci (Figures 9B,B′). The comparison of the subcellular localization for each transgelin protein in the gut circumferential muscles revealed a clear difference: Mp20-sfGFP was colocalized with F-actin only in the Z-lines of the visceral muscles, while CG5023-YFP was colocalized with F-actin along the entire sarcomere unit (Figures 9A,B), like in the larval stage (Figures 5G,H). CG5023-YFP was also detected within the muscle nuclei (Figures 9B,B′). The underlining enterocytes of the gut accommodated high levels of Chd64-GFP not only in the cytoplasm but also in the nucleus (Figures 9C,C′).

Lastly, we examined the expression pattern of fly transgelins in both female and male genitalia. Both Mp20-sfGFP and CG5023-YFP were similarly expressed in the muscle sheath surrounding the developing egg chambers in the female ovarioles (Figures 9D,E), the muscle sheath surrounding the ejaculatory duct (Figures 9K,L,N,N′), the accessory gland (Figures 9K,L,O,O′), the smooth muscles surrounding the testes (Figures 9K,L,P,P′), and the muscle fibers of the ejaculatory bulb (Figures 9K,L,Q; Susic-Jung et al., 2012). Interestingly, in the muscles surrounding the egg chambers, both Mp20-GFP and CG5023-YFP had an identical subcellular distribution in sarcomeres and the nuclei (Figures 9D,E,G,G′,H,H′). Chd64-GFP was expressed in follicular epithelial cells (Figures 9F,I,I′) and displayed a specific higher expression in the polar cells (Figures 9F,I,I′). In males, Chd64-GFP was expressed in all anatomical parts that form the genitalia, including the somatic cells encapsulating the germline in the testes (Figures 9M,R,R′), the ejaculatory bulb (Figure 9M), and the binucleated accessory gland epithelium (Figures 9S,S′,P,P′). In the latter epithelium, Chd64 was localized in both cytoplasmic granules and the nuclei (Figures 9S,S′).

## Discussion

### The Small Actin-Binding Motif Is Not Conserved in *Drosophila* Transgelins

In this study, we have generated and characterized appropriate genetic and molecular tools to thoroughly investigate the protein expression and tissue distribution of the three *Drosophila* transgelins, namely, Mp20 (CG4696), CG5023, and Chd64 (CG14996) in a living organism. Mammalian transgelins comprise a conserved family of actin-binding proteins (Shapland et al., 1993; Fu et al., 2000; Mori et al., 2004; Na et al., 2015), and their general domain organization is that of a single N-terminal CH-domain and a single C-terminal CLR. The latter region has been shown to mediate the interaction of human TAGLN with actin (Fu et al., 2000). Recently, a second ABM was identified in mouse TAGLN2 to be essential in stabilizing F-actin (Na et al., 2015), supporting previous elegant biochemical studies in the yeast homolog of transgelin SCP1 that had predicted the existence of a second actin-binding site between the CH-domain and the CLR (Goodman et al., 2003). Here, we report that this small ABM is not conserved in all three *Drosophila* transgelins. Taking into account previous biochemical fractionation of larval body wall muscle lysate that showed weak association of Mp20 with the myofibrils (Ayme-Southgate et al., 1989), it remains unclear how *Drosophila* transgelins associate directly with intracellular actin filaments.

### Mp20 and CG5023 Display Both Common and Unique Expression in Muscle Tissues During Development

**FIGURE 10:**
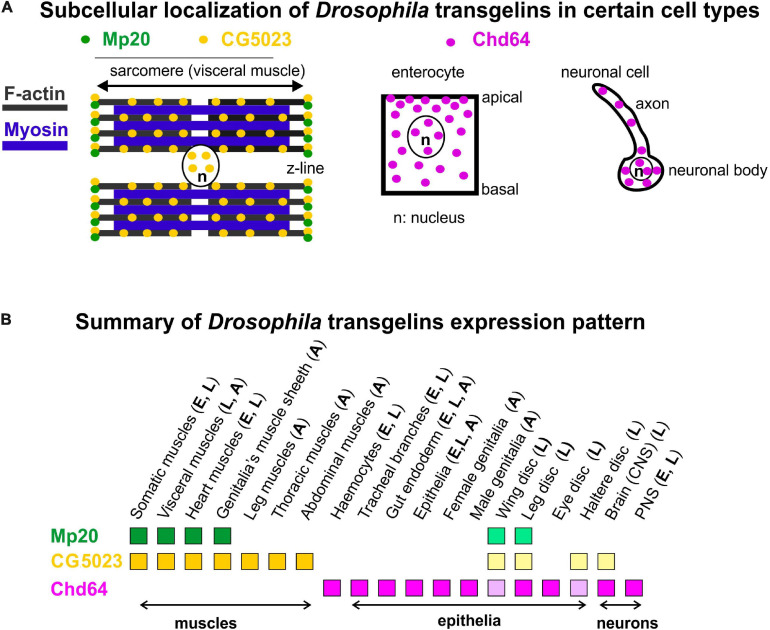
**(A,B)** Collective schematic representation of **(A)** subcellular transgelins localization in specific cell types (visceral muscle for Mp20 and CG5023; enterocyte and neuronal cell for Chd64) and **(B)** tissue expression pattern throughout fly development.

Our data provide direct evidence that most of the muscle tissues in *Drosophila* contain two transgelin proteins: Mp20 and CG5023 (Figures 10A,B). However, there are certain deviations from this situation that indicate unique muscle type-specific functional requirement. First, we confirmed the high expression levels of Mp20-GFP protein in a subset of embryonic muscles, including VA2, VA3, SBM, and DT1. The enrichment of Mp20 in these muscles has been linked with its modulatory role in the fusion process. RNAi-mediated knockdown of Mp20 in embryonic somatic muscles resulted in unfused myoblasts (Bataillé et al., 2010). A similar regulatory function for the fusion process could also be attributed to CG5023 because it is highly accumulated within the DO1–2/DA1–2 embryonic muscles of the thoracic segments. Second, CG5023 can be detected earlier in the developing dorsal vessel in the embryo. Third, CG5023 is uniquely expressed in the alary muscles, which are multinucleated sarcomeric muscles that maintain the position of the internal larval organs (Bataillé et al., 2020). Fourth, Mp20 and CG5023 have a complementary expression in the larval and adult gut’s circumferential muscles. Mp20 is accumulated mainly in the larval vmlo, while in the adult, it is expressed at both vmlo and vmci. On the contrary, CG5023 is expressed only in the vmci. Fifth, the subcellular localization of Mp20 and CG5023 is quite distinct in the gut circumferential muscles. Mp20 labels only the myotubes’ Z-lines (Ayme-Southgate et al., 1989). CG5023 is localized in a striated pattern and is also strongly detected in the nucleus (Figure 10A). It remains unclear whether the tight colocalization of CG5023 with F-actin in the vmci is related to the presence of the two positive charged residues (K^128^ and R^131^) in the small ABM, which otherwise is poorly conserved overall as previously mentioned. Sixth, CG5023 is the only transgelin protein detected in the adult body wall somatic musculature, including abdominal muscles, synchronous muscles in the thorax, pharyngeal muscles, and leg muscles. This was a very surprising finding given the previous report of detecting Mp20 in the adult thoracic synchronous muscles by Western blotting (Ayme-Southgate et al., 1989). However, the adult expression of CG5023 in the thoracic muscles is consistent with the RNAi-mediated knockdown studies, which resulted in flightless adult flies (Schnorrer et al., 2010). Finally, we discovered that only CG5023 is expressed in few neuronal cells in the larval brain lobes and vnc, exemplifying the only non-muscle tissue of its expression.

### Endodermal and Ectodermal Chd64 Expression

Chd64 developmental expression pattern mostly resembles the expression pattern of mammalian TAGLN2 and TAGLN3, while the expression pattern of Mp20 and CG5023 resembles more the expression pattern of TAGLN (Figure 10B). High accumulation of Chd64 in embryos and larvae is observed in a variety of cell types that need to coordinate in an actin-dependent manner (a) their integrity (e.g., gut epithelium and tendon cell of epidermis), (b) migratory properties (e.g., final branches of trachea and hemocytes), and (c) axonal transport (e.g., larval nerves). For example, Chd64 was detected at significantly high levels in the secondary and final tracheal branches. These are structures that undergo dynamic cell shape changes during their formation in embryo and their remodeling in larva to meet the needs of tissues in oxygen supply. On the other hand, Chd64 protein levels were low in neuronal cell types in the embryo and sharply were increased later in larvae. Previous studies have shown that mammalian TAGLN3 optimal level is required for maximal neurite growth (Pape et al., 2008). Perhaps, this temporal regulation of Chd64 expression may be related to its functional requirement in the precise adjustment of actin filament dynamics that modulate axonal transport and synaptic transmission (Konietzny et al., 2017). The expression of Chd64 in the epithelium encapsulating the developing gametes in both female and male genitalia implies a potential role for Chd64 in the adult fly fertility, in agreement with the proposed role of TAGLN2 in the mouse blastocyst and embryo implantation (Liang et al., 2019).

Chd64 protein displays a dual subcellular distribution in the cytoplasm and the nucleus (Figure 10A). While the cytoplasmic localization of Chd64 could be linked with its putative role in the modulation of actin cytoskeleton, its nuclear presence fits with previous studies reporting binding of Chd64 on Juvenile hormone receptor element 1 (JHRE1) and ecdysone response element (EcRE) (Li et al., 2007), suggesting a putative role of *Chd64* in transcription modulation. Interestingly, structural analysis indicated that Chd64 is a pliable protein-containing terminal intrinsically disordered regions (IDRs) that facilitate multiple molecular interactions (Kozłowska et al., 2014; Tarczewska et al., 2015). Our data clearly demonstrate a complete absence of Chd64 expression in muscles during fly development. However, the available UAS:RNAi lines (GD1212 and KK102197) for Chd64 upon expression with *mef2Gal4* generated larval lethality, likely due to off-target effect of other essential gene(s) for muscle function (Schnorrer et al., 2010). Thus, currently, we lack specific tools to address the functional requirement of Chd64 in *Drosophila*, but specific null mutants will undoubtedly provide this information.

In conclusion, here we report for the first time a detailed expression profile of all transgelins throughout *Drosophila* development. From our analysis, we predict that Mp20 and CG5023 most likely functionally synergize within muscles, while Chd64 mediates unique functions in endodermal and ectodermal tissues.

## Materials and Methods

### *Drosophila* Genetics

GFP-tagged flyfos TransgeneOme (fTRG) lines for CG5023 (318216) and Mp20 (318119) were obtained from VDRC (Sarov et al., 2016). Other stocks used in this study include *UAS:ABD-Moesin-RFP* (T. Millard), *btl-Gal4*, and *UAS:GFP*/CyO (Ghabrial et al., 2011). Embryos bearing the third chromosome lesion *Df(3L)ens^Δ^^3277^*, *Chd64^Δ^^3277^ens^Δ^^3277^P[neoFRT]80B/TM3*, *Sb* (BL-51319) were used for the characterization of the Chd64 antibody. All other stocks were obtained from Bloomington Stock Center. All crosses were performed at 25°C.

### Generation of Mp20-GFP, CG5023-YFP, and Chd64-GFP Transgenes

Mp20-GFP: BAC clone R05M17 was initially digested with *Stu*I*/Bsp*EI. The 8.8-kb genomic fragment bearing −5.6/+0.6 kb flanking Mp20 gene sequence was subcloned into *pBluescript*. A *Bcl*I*/Bgl*II 2-kb Mp20 3′-fragment region was fused with a four Ser-linker in frame with eGFP. The full-length *Eco*RV/*Bgl*II Mp20-GFP-engineered sequence was finally cloned in the P-element transformation vector *pCaspR3*. CG5023-YFP: BAC clone R09G02 was digested with *Xba*I/*Bam*HI, and the 14.2-kb genomic fragment bearing −5.3/+3.8 kb flanking CG5023 gene sequence was subcloned into *pSL1180*. The *Sap*I-flanking 2.2-kb CG5023 3′-fragment was fused with a four Ser-linker in frame with vYFP. The full-length *Xba*I/*Bam*HI CG5023-YFP-engineered sequence was finally cloned in the P-element transformation vector *pCaspR3*. Chd64-GFP: BAC clone R48M07 was digested with *Avr*II and the 9.9-kb genomic fragment bearing −0.4/+1.4 kb flanking Chd64 gene sequence was subcloned into *pBluescript*. An *Spe*I/*Xho*I 2.4-kb Chd64 3′-fragment was fused with a four Ser-linker in frame with eGFP. The full-length Chd64-GFP-engineered sequence was finally cloned in P-element transformation vector *pCaspR3*. At least three transgenic lines were obtained and analyzed for each of the three genes.

### Generation of Anti-Chd64 Antibody and Western Blotting

A polyclonal antibody was generated using the His-tagged fusion protein corresponding to full-length Chd64 amino acids 1–188. *Bam*HI/*Hin*dIII fragment of *Chd64* cDNA clone GH28730 was fused into *pET28b*(+) (Novagen, Madison, WI, United States). The expression and purification of the recombinant protein was performed according to the manufacturer’s recommendations. Antibody specificity was tested by Western blotting and immunohistochemistry on either wild-type embryos or embryos deficient for Chd64 protein expression. Protein lysates were prepared from late-stage embryo or adult flies and analyzed by Western blotting with antibodies against Chd and Parvin as a loading control and developed as previously described (Vakaloglou et al., 2012).

### Embryonic Sample Preparation

Embryos were collected from timed egg-lays and appropriately staged at 25°C (Campos-Ortega and Hartenstein, 1985) prior to whole sample preparation. Whole embryo preparation fixations were mostly performed in 4% formaldehyde in PBS; alternative fixation treatment included 90% methanol. PBT (0.5% BSA and 0.2% Triton X-100 in PBS) was used for blocking, washes, and primary and secondary antibody incubation either at room temperature or at 4°C.

### Larval and Adult Tissue Sample Preparation

Late third-instar larvae were dissected in PBS to isolate imaginal disks and the gastrointestinal tracts that were subsequently fixed with 4% formaldehyde in PBS according to standard protocols. Dissection, immunostaining, and mounting samples of the adult gastrointestinal tract were performed as described by Micchelli (2014). Adult ovarian and testicular tissue samples were isolated, fixed, and processed according to Thompson et al. (2015).

### Immunofluorescence Experiments and Microscopy

Primary antibodies used in this study were against the following: Chd64 (rabbit polyclonal anti-serum; 1:1,000), MHC (myosin heavy chain, mouse monoclonal; 1:60) (Kiehart et al., 1990), FasII (mouse monoclonal clone 1D4 from DSHB) to visualize motor neurons (Lin et al., 1994), FasIII (mouse monoclonal clone 7G10 from DSHB), Dac (mouse monoclonal clone mAbdac1-1 from DSHB, a kind gift from Tasos Pavlopoulos at IMBB-FoRTH), and Elav (rat monoclonal clone 7E8A10 from DSHB, a kind gift from Christos Delidakis at IMBB-FoRTH). Species-specific secondary antibodies used were conjugated with Alexa Fluor 568 or 633 (Molecular Probes, Eugene, OR, United States; Life Technologies, Carlsbad, CA, United States) diluted at 1:1,000. Nuclei were labeled with DAPI. F-actin was visualized using either rhodamine-phalloidin or Alexa Fluor 647-phalloidin at 1:500 dilution (Molecular Probes; Life Technologies, Carlsbad, CA, United States). All samples were mounted in Vectashield medium (Vector Laboratories, Burlingame, CA, United States).

Single confocal sections and *z* stacks were acquired on a Leica TCS SP5 laser scanning inverted confocal microscope with an HC Plan Apochromat × 20/0.7 or HC Plan Apochromat × 63/1.4 oil objective. Whole adult fly imaging was performed with an HC Plan Apochromat × 5/0.15 objective at a maximum zoom of 1.5. Confocal settings were adjusted to avoid pixel intensity saturation of 1,024 × 1,024 pixel images captured at 400 Hz. Postacquisition assembly was performed with LAS AF software (v.2.3.6). For live monitoring, *z* stacks imaged over time consist of 12 single focal sections of 2 μm and 1,024 × 512 pixels, captured at 400 Hz and recorded at 5 min intervals for 2–3 h. GFP was excited with the 488-nm argon laser line and emission was recorded in the 495–525-nm range. Individual focal planes for each time point were selected using LAS AS software (v 2.3.6) and then processed and converted into movies (12 frames/s) using ImageJ. For the quantification of fluorescent amount of transgelins among tissues, maximal projections of confocal stacks were produced. Mean value of fluorescence intensity of manually selected areas of the same size was quantified using ImageJ software. All images were processed using Photoshop 7 and labeled in CorelDRAW 12.

## Data Availability Statement

The raw data supporting the conclusion of this article will be made available by the authors, without undue reservation.

## Author Contributions

KV, MM, AK, and CZ: conceptualization, methodology, and investigation. CZ: writing-original draft. KV, AK, and CZ: writing-review and editing. CZ and AK: funding acquisition. CZ: supervision. All authors contributed to the article and approved the submitted version.

## Conflict of Interest

The authors declare that the research was conducted in the absence of any commercial or financial relationships that could be construed as a potential conflict of interest.

## Footnotes

1 http://nls-mapper.iab.keio.ac.jp

## Supplementary Material

Supplementary Figure 1 **(A,B)** Protein sequence alignment among the three *Drosophila* transgelins and their mouse homologs to confirm: **(A)** conservation between Calponin Homology (CH) domain, C-terminal calponin-like repeat (CLR) regions and the predicted bipartite nuclear localization signal (NLS) based on an algorithm described in Kosugi et al. (2009) available in the cNLS Mapper website (http://nls-mapper.iab.keio.ac.jp). The putative NLS highlighted with a dotted black box in each protein sequence. **(B)** *Drosophila* transgelins lack of conservation within the small Actin Binding Motif located in between CH-domain and CLR.

Supplementary Figure 2 Live-imaging of embryo expressing CG5023-sfGFP (lateral view) showing accumulation in migrating myoblasts.
