# Supplementary figures and images for: Differential Expression of Drosophila Transgelins Throughout Development
Source: Front Cell Dev Biol. 2021 Jul 12;9:648568. doi: 10.3389/fcell.2021.648568 (PMC8311604; doi:10.3389/fcell.2021.648568)

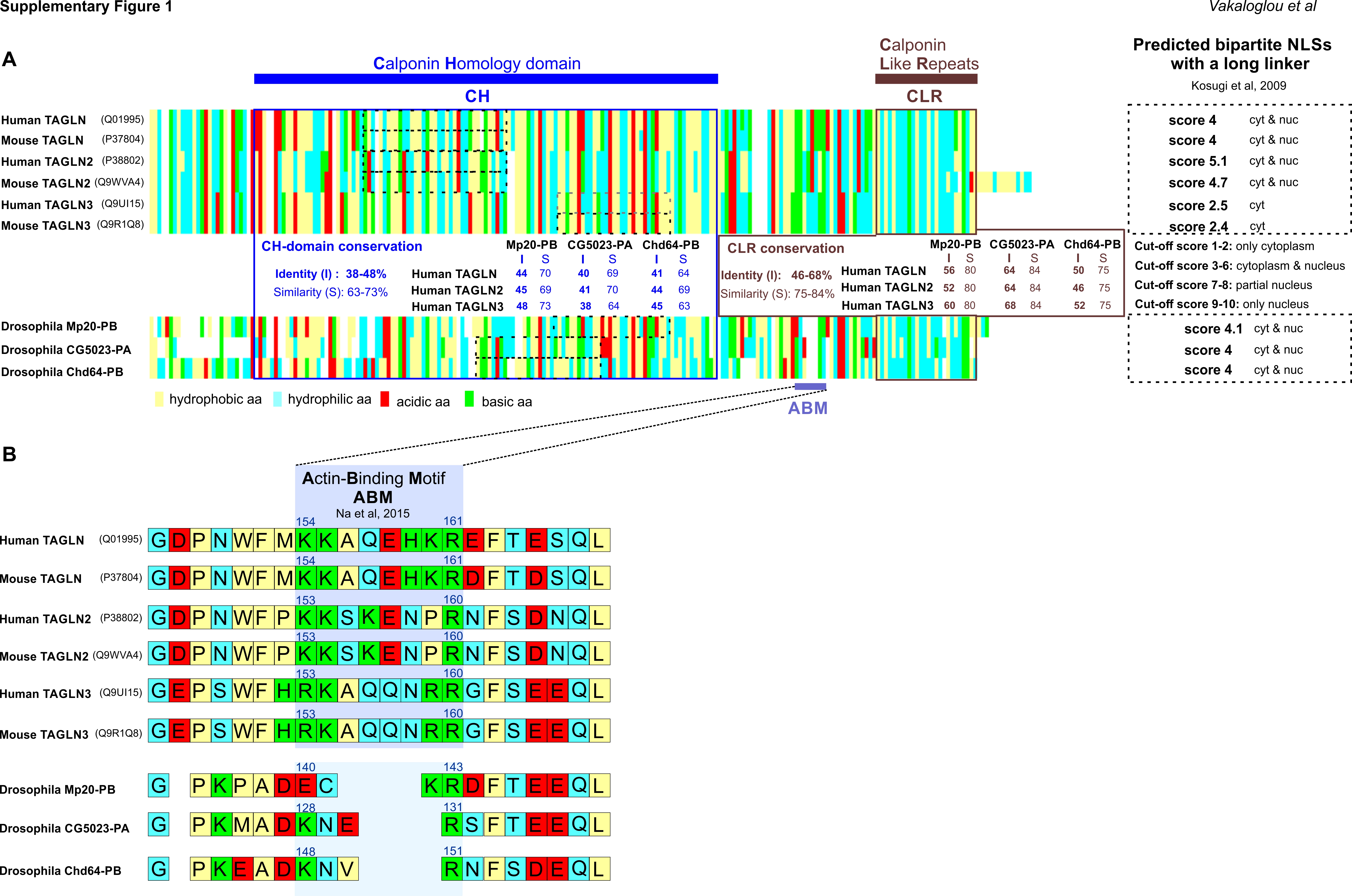

Supplement: Supplementary Figure 1 — (A,B) Protein sequence alignment among the three Drosophila transgelins and their mouse homologs to confirm: (A) conservation between Calponin Homology (CH) domain, C-terminal calponin-like repeat (CLR) regions and the predicted bipartite nuclear localization signal (NLS) based on an algorithm described in Kosugi et al. (2009) available in the cNLS Mapper website (http://nls-mapper.iab.keio.ac.jp). The putative NLS highlighted with a dotted black box in each protein sequence. (B) Drosophila transgelins lack of conservation within the small Actin Binding Motif located in between CH-domain and CLR. [file Image_1.JPEG]
